# Dynamic neural states underpin motor symptom severity in Parkinson's disease: a longitudinal analysis of chronic cortico-subthalamic nucleus recordings

**DOI:** 10.1016/j.ebiom.2026.106293

**Published:** 2026-05-12

**Authors:** Abhinav Sharma, Tao Liu, Bahman Abdi-Sargezeh, Amelia Hahn, Maria Shcherbakova, Wolf-Julian Neumann, Simon Little, Philip Starr, Ashwini Oswal

**Affiliations:** a Nuffield Department of Clinical Neurosciences, University of Oxford, Oxford, United Kingdom; b MRC Brain Networks Dynamics Unit, University of Oxford, Oxford, United Kingdom; c Weill Institute for Neurosciences, Department of Neurology, University of California San Francisco, San Francisco, CA, USA; d Movement Disorder and Neuromodulation Unit, Department of Neurology, Charité – Universitätsmedizin, Berlin, Germany

**Keywords:** Neural states, Bradykinesia, Parkinson's disease

## Abstract

**Background:**

Motor symptoms in Parkinson's disease (PD) may arise due to transient, network-wide neural dynamics that extend beyond beta-band oscillatory activity within the motor cortical-subthalamic nucleus (STN) circuit.

**Methods:**

We applied a four-state Hidden Markov Model (HMM) to identify states of local and interregional oscillatory synchrony from chronic motor cortical and STN recordings (1046 h from 10 hemispheres) in five patients with PD (mean age 49 years), with concurrent measurements of bradykinesia, dyskinesia and tremor quantified using wearable sensors.

**Findings:**

Neural states exhibited distinct spectral and temporal features relating to symptom severity. Two states exhibited spectral signatures—particularly STN low and high gamma, STN delta/alpha, cortical beta, and cortico-STN beta coherence—that predicted worsening bradykinesia. STN beta oscillations were not consistent predictors of bradykinesia (p = 0.52), but did predict worsening tremor (p < 0.01) and also improvements in dyskinesia severity (p < 0.001), in a state specific manner. These states also displayed compensatory features associated with bradykinesia amelioration, including cortical delta/alpha activity, cortical high gamma, and cortico-STN high gamma coherence. Additionally, we identified a state, marked by STN beta without cortico-STN beta coherence, whose increased lifetimes and occurrence improved motor function (p < 0.001).

**Interpretation:**

Our findings highlight the multidimensional nature of motor impairments in PD and suggest that adaptive interventions targeting state features—rather than single frequency bands—may offer new opportunities for personalised neuromodulation.

**Funding:**

AO: MRC Clinician Scientist Fellowship (MR/W024810/1), Rosetrees Trust/Race Against Dementia Team award, Oxford Hospitals Charity, and the Jon Moulton Charity Trust. TL: China Scholarship Council.

**Research in context**

**Evidence before this study**

We searched the PubMed database (articles from database inception to 10th February 2026) for studies linking subthalamic nucleus (STN) oscillatory activity to Parkinson's disease motor symptoms. These studies consistently demonstrate that STN oscillatory activity at beta (15–30 Hz) frequencies correlates positively with motor symptoms over long timescales in Parkinson's disease. This observation has led to beta activity being used as a biomarker for adaptive Deep Brain Stimulation. It remains unclear however whether other oscillatory features within the motor cortical-subthalamic nucleus network could provide improved biomarkers for dynamically tracking symptom severity.

**Added value of this study**

We address this by performing chronic motor cortical and subthalamic nucleus recordings in patients with Parkinson's disease during activities of daily living. Simultaneous measurements of symptom severity were captured using wearable sensors. We used Hidden Markov Models to identify transient states of neural activity and related these to symptom severity. Although cortical beta and cortico-STN beta coherence predicted worsening motor symptoms, STN beta activity alone was not a consistent predictor. We identified spectral features associated with motor symptom improvements, including cortical delta/alpha activity, cortical high gamma, and cortico-STN high gamma coherence. Additionally, there was a compensatory state characterised by short-lived cortical and subthalamic nucleus beta activity, whose increased occurrence was associated with symptomatic improvements.

**Implications of all the available evidence**

Our findings highlight the importance of prolonged, high temporal resolution measurements of both neural activity and symptom severity for discovering adaptive Deep Brain Stimulation biomarkers. Crucially, we identify new target states and spectral features for improving motor symptoms in Parkinson's disease.

## Introduction

Bradykinesia is a cardinal motor symptom of Parkinson's disease (PD), characterized by a pathological reduction in movement velocity and amplitude, significantly impairing patients' quality of life.^1^, ^2^, ^3^ From a therapeutic perspective, bradykinesia exhibits a robust response to dopaminergic therapy and high-frequency deep brain stimulation (DBS).^4^, ^5^, ^6^ Mechanistically, beta-band oscillatory activity (12–30 Hz) within the subthalamic nucleus (STN) has been extensively investigated as a neurophysiological correlate of bradykinesia, often assessed through the motor subsection of the Unified Parkinson's Disease Rating Scale (UPDRS-III).^7^, ^8^, ^9^, ^10^, ^11^, ^12^, ^13^, ^14^, ^15^ At the network level, increased coherence between the motor cortex and STN within the beta frequency range has been implicated in akinetic-rigid manifestations of PD, suggesting a circuit-wide pathophysiological mechanism.^16^^,^^17^ Notably, cortical beta activity, particularly within primary motor regions, has also been linked to worsening motor deficits in PD, reinforcing the hypothesis that exaggerated beta synchrony contributes to impaired motor function.^18^, ^19^, ^20^, ^21^, ^22^ Both dopaminergic medication and DBS can attenuate beta oscillatory activity within the cortex and STN, while concurrently reducing cortico-subthalamic synchrony. These neurophysiological changes closely correlate with improvements in motor function, establishing a strong association between pathological beta synchronization and bradykinesia.^10^^,^^23^, ^24^, ^25^, ^26^, ^27^

Previous research has increasingly recognised the pathological significance of oscillatory dynamics beyond the beta band, implicating multiple frequency domains in the pathophysiology of PD.^28^^,^^29^ Furthermore, some evidence also challenges the traditional view that beta oscillations alone drive bradykinesia, as experimental manipulations of beta-band activity in animal models do not consistently exacerbate motor impairment.^30^, ^31^, ^32^ Recently it has been proposed that low-frequency rhythms in the delta (∼2–4 Hz) and theta (∼4–10 Hz) bands contribute to motor dysfunction. Exaggerated low-frequency oscillations in the STN have been observed in dopamine-depleted states,^33^ with correlations to both bradykinesia and the occurrence of tremor.^33^, ^34^, ^35^

In the higher frequency domain, gamma oscillations (40–100 Hz) have been consistently identified as exerting a prokinetic influence in PD. Investigations into gamma–band activity typically involve spectral power analyses in key regions, notably the STN and motor cortex, as well as assessments of cortico-subthalamic coherence. These measures are frequently correlated with UPDRS-III motor scores under dopaminergic medication conditions.^36^, ^37^, ^38^ Task-specific studies reinforce the prokinetic role of gamma oscillations, demonstrating their positive association with movement velocity, vigour, grip force, and their augmentation in response to levodopa administration.^39^, ^40^, ^41^, ^42^ Mechanistically, gamma activity may function in direct opposition to beta oscillations in PD. This dynamic interplay suggests that an optimized motor state, characterized by diminished bradykinesia and rigidity, emerges when beta-band synchrony is suppressed, concomitant with an upregulation of gamma oscillations within the STN or the STN-motor cortex network.^39^^,^^41^ In keeping with this hypothesis is the observation that dyskinesias, which are involuntary movements triggered by chronic dopaminergic medication use, are frequently associated with excessive gamma activity within the STN-motor cortex network.^36^

The motivation for this study stems from the growing realization that parkinsonian motor symptoms do not arise from a single, uniform neural substrate, but rather from dynamically fluctuating and spatially distributed circuit states involving diverse oscillatory regimes. While previous studies have established broad associations between frequency bands and motor symptoms, a central paradox in Parkinsonian physiology is that similar clinical manifestations may arise from distinct, and sometimes even opposing, spectral configurations. Beta synchrony may co-occur with compensatory gamma bursts, and delta oscillations may mark either a persistent akinetic state or transiently decouple during movement. This raises three fundamental mechanistic questions that static spectral analysis cannot address.^43^^,^^44^ First, do alterations across different spectral bands occur simultaneously, or are they temporally segregated into distinct network states? Second, are pathological changes confined to localized regions such as the STN, or do they require circuit-wide propagation through cortico-subthalamic coherence? Third, which specific state-resolved neural features serve as the most reliable predictors of bradykinesia, dyskinesia, and tremor severity? Resolving these questions requires moving beyond temporal averaging to characterize how the motor network dynamically organizes itself across time.

To address these questions, we adopt a probabilistic framework grounded in Hidden Markov Models (HMMs) to identify recurring spatiotemporal patterns or states, within continuous neural recordings.^43^ These states capture network configurations characterized by distinct covariance structures encompassing STN and motor cortical activity, as well as inter-regional coherence across canonical frequency bands. Crucially, this approach allows us to test whether motor symptom (bradykinesia, dyskinesia and tremor) related spectral features co-occur within the same temporal epoch or emerge in dissociable states, whether pathology manifests as local power changes or requires circuit-level synchronization, and which state-specific properties most reliably predict symptom severity. We apply this framework to chronic cortico-STN recordings from DBS devices, paired with continuous objective measurements of bradykinesia, dyskinesia, and tremor from wearable sensors during naturalistic behaviour. This approach represents a shift away from one-dimensional biomarkers toward a multidimensional understanding of neural state architecture in Parkinson's disease that could inform the next generation of personalised therapeutic interventions.

## Methods

### Patient cohort and electrode localization

We analysed data from five individuals (4 males, 1 female) diagnosed with Parkinson's disease (PD), each implanted bilaterally with the Medtronic Summit RC + S neural interface. Subthalamic nucleus (STN) recordings were acquired via quadripolar Medtronic 3389 electrodes, while cortical signals were recorded using quadripolar paddle-type leads with 10 mm inter-contact spacing. The cortical leads were positioned to ensure that 2–3 contacts lay anterior to the central sulcus and approximately 2–4 cm lateral to the midline. Electrode localization was verified using a linear co-registration pipeline that fused postoperative CT with preoperative 3T MRI, following previously validated protocols.^36^^,^^45^ Electrode contact coordinates were normalized to Montreal Neurological Institute (MNI) space using the Lead-DBS toolbox^46^ for group-level visualisation (Supplementary Figure S1).

Neural signals were streamed at a sampling rate of 250 Hz from both hemispheres to a Microsoft Windows tablet. Bipolar recordings were streamed from STN contacts 1–3 and 2–4 (numbers ordered from inferior to superior) and from cortical contacts 1–3 and 2–4 (numbers ordered from anterior to posterior). At least one STN contact from each bipolar channel was placed within the sensorimotor subregion of the STN, and one cortical contact overlayed the motor cortex (Supplementary Figure S1). Patients initiated home recordings were streamed to the Microsoft tablet in 1–2 week recording sprints (see Supplementary Methods and Gilron et al., 2021^45^).

Recordings were acquired 2–4 weeks post-surgery while patients performed naturalistic behaviours in their home environment, while taking their regular dopaminergic medication, prior to deep brain stimulation (DBS) initiation. Patients were instructed to continue their normal daily activities, including active behaviours (e.g., conversations, household tasks), passive activities (e.g., watching television, reading), postural transitions, eating, personal care, and periods of rest. Clinical characteristics of each patient are provided in Table 1. Concurrent motor symptom data were collected using bilateral wrist worn Personal KinetiGraph® (PKG®) monitors (Global Kinetics Pty Ltd.), which provided continuous 2-min interval assessments of bradykinesia, tremor, and dyskinesia, based on validated criteria.^45^

**Table 1:** Clinical characteristics of patients.

Patient	Age, sex, and ethnicity	Disease duration (years)	Preoperative medication (mg)	UPDRS III off medication	% Change in UPDRS III when on	Off medication tremor scores	MOCA	Data duration with concurrent contralateral PKG. Durations of tremor and dyskinesia are also shown (hours)
1 02	54 M White, Caucasian	7	LDE 1425	49	90%	L: 2 R: 0	26	L: 123.7 (tremor = 2.7; dyskinesia = 23.3) R: 192.9 (tremor = 2.9; dyskinesia = 40.7)
2 05	63 M Hispanic or Latino	19	LDE 955	45	51%	L: 3 R: 4	30	L: 27.7 (tremor = 1.2; dyskinesia = 5.3) R: 24.0 (tremor = 0.5; dyskinesia = 4.2)
3 06	28 F Hispanic or Latino	12	LDE 1550	61	73%	L: 12 R: 9	27	L: 80.5 (tremor = 5.7; dyskinesia = 17.2) R: 162.7 (tremor = 12.5; dyskinesia = 35.8)
4 07	40 M White, Caucasian	4	LDE 1314	41	65%	L: 6 R: 3	30	L: 192.7 (tremor = 23.6; dyskinesia = 36.1) R: 175.6 (tremor = 32.6; dyskinesia = 30.4)
5 08	58 M White, Caucasian	12	LDE 2100	44	75%	L: 0 R: 0	27	L: 40.4 (tremor = 2; dyskinesia = 6) R: 25.7 (tremor = 0.2; dyskinesia = 0.5)

LDE, levodopa dose equivalent; MOCA, Montreal Cognitive Assessment.

The total pre-operative Movement Disorder Society-Unified Parkinson's Disease Rating Scale (UPDRS) part III score is presented in the off-medication state. Off-medication tremor scores (sum of UPDRS part III items 16a to 17d) are also shown separately for each hemisphere to indicate the extent of tremor.

### Hidden Markov Model fit

A single Hidden Markov Model (HMM) was trained on the longitudinal neural recordings from all five patients. Each input file corresponded to one hemisphere and included four bipolar channels, two from the motor cortex and two from the STN. Files were excluded if any channel lacked valid signal or contained non-contiguous segments; in such cases, continuous fragments were isolated for analysis (see Methods: Patient Cohort and Electrode Localization). To reduce the influence of amplitude variability across channels, each channel was separately subjected to percentile normalization across the full dataset.

Time-delay embedding was applied using a symmetric 15-point window (−7:0:+7), transforming the 4-channel input into a 60-dimensional vector per time point (4 channels × 15 delays), spanning 60 ms at 250 Hz. Signals were bandpass filtered (2–120 Hz) to retain physiologically relevant oscillatory dynamics. The resulting data matrix (60 × time) for each session was used as input to the HMM, implemented using the Python version of the OSL-dynamics toolbox^47^ (https://osl-dynamics.readthedocs.io/en/latest/index.html). Each latent state was modelled using multivariate Gaussian emissions, initialized with random mean vectors and full-rank covariance matrices. Latent states identified by the HMM each represent a recurring configuration of neural activity characterised by a distinct pattern of oscillatory power and coherence across brain regions. The HMM probabilistically identifies these configurations by detecting when the statistical structure of multi-channel recordings exhibits similar covariance patterns. The covariance matrices inferred from the estimation regime capture all relevant spectral features for each state. This covariance includes not just instantaneous correlations between regions (e.g., STN and cortex), but also cross-frequency phase relationships and time-lagged interactions that reflect both local rhythmicity and large-scale coupling patterns.^43^^,^^48^ For instance, a given state might be defined by strong cortico-subthalamic coherence at beta frequency, STN-local autocovariance at a gamma timescale, or some hybrid of both. As long as these covariance features persist, the state identity remains intact, even if absolute spectral power fluctuates.

Crucially, this formulation enables a unified characterization of both local and circuit-level dynamics within a single probabilistic framework. Rather than treating power and coherence as separate variables or applying windowed averages, the TDE-HMM integrates them into a joint representation. This allows us to dissect how transient shifts in network-level synchrony or regional oscillatory bursts contribute to motor symptoms without collapsing them into a single frequency bin. Models with 3, 4, and 6 states were evaluated, and the 4-state model was selected for downstream analyses (see Spectral Characterization of HMM States for justification).

### PKG® alignment and preprocessing of behavioral data

PKG® bradykinesia scores were temporally aligned with neural recordings using timestamp synchronization.^22^^,^^45^ Each PKG® score corresponded to a 2-min segment of neural data, defining the analysis window. For contralateral alignment, neural data from one hemisphere were paired with PKG® scores from the opposite wrist.

Bradykinesia scores were transformed to positive values for consistency. This meant that a higher bradykinesia score indicates worse bradykinesia in the patient. Epochs associated with prolonged immobility (duration >2 min and bradykinesia >80) were classified as sleep and excluded as per previous analyses.^22^^,^^45^^,^^49^ For bradykinesia analysis, we excluded epochs where bradykinesia scores were below each patient's 30th percentile across the full recording period.^36^^,^^45^ This allowed us focus on data segments that captured a broad range of variance in bradykinesia scores, without including data that would be confounded by the presence of on state dyskinesias. In contrast, for dyskinesia analysis we focused our analysis on epochs—corresponding to on medication states^36^^,^^45^–where bradykinesia scores were below the 30th percentile. Finally for relating neural features to tremor severity, we included data segments during which tremor was present (indicated by PKG® tremor scores above 0).

### Static spectral analysis

For each 2-min window, we employed Welch's method to calculate auto-power spectral densities (PSDs) for individual brain regions (STN and motor cortex) and cross-power spectral densities between regions. We applied a 50% overlap between analysis windows with a window length of 2 s and a Hanning windowing function, yielding a frequency resolution of 0.5 Hz across the 2–100 Hz frequency range. This frequency resolution was consistent with our spectral analysis of HMM states. Magnitude squared coherence between STN, and motor cortex was calculated from the auto- and cross-PSDs.

### Spectral characterization of HMM states

State time courses were extracted from the trained HMM, yielding continuous-valued probabilities (range: 0–1) representing the instantaneous likelihood of each state being active. These were subsequently binarized using the Viterbi algorithm to obtain discrete state assignments.

Within each 2-min window, state-specific neural data were isolated by element-wise multiplication of binarized state time courses with the corresponding non-embedded raw signals. Power spectral density (PSD) and coherence were computed using multi-taper spectral analysis with seven Slepian tapers (time-bandwidth product of 4, frequency resolution of 0.5 Hz) and 50% overlapping windows.

For each state, PSDs were averaged across STN channels to produce a single STN PSD, and across cortical channels to yield a CTX PSD. Coherence was computed for all STN–CTX channel pairs and averaged to derive a single STN–CTX coherence value per state. These features together formed the combined spectral and coherence fingerprint of each latent brain state. Our rationale for averaging was motivated by the spatially overlapping configuration of bipolar recording channels at each site (see Methods under the heading Patient Cohort and Electrode Localisation).

In the standard OSL-dynamics pipeline, spectral outputs are often subjected to non-negative matrix factorization (NNMF) to reduce noise artifacts that arise due to limited sample sizes, a problem exacerbated by models with a large number of states or spatial channels. In our dataset, spatial dimensionality was not a limiting factor. However, training a six-state HMM degraded spectral estimation quality to the extent that NNMF became necessary. Because NNMF introduces additional complexity and requires parameter tuning, we opted for a four-state model. This reduction significantly improved the spectral clarity of states and obviated the need for NNMF, thereby simplifying the pipeline and enhancing interpretability for mechanistic clinical inference.

### Temporal metrics of HMM states

Discrete state time courses allowed the calculation of several temporal features within each 2-min analysis window:

• Fractional Occupancy (FO): Proportion of time each state was active.
• State Lifetime: Average duration of continuous occupancy of a given state.
• Visit Interval: Mean interval between successive activations of the same state.

These metrics collectively characterized the temporal properties of latent brain states.

### Modelling symptom severity using static spectral features

To establish the relationship between static spectral features and bradykinesia, dyskinesia or tremor severity, we employed a generalized linear model (GLM) framework with 2-min time windows as units of analysis. For each window, we extracted 15 spatio-spectral features, comprising three spatial metrics (STN PSD, CTX PSD, and STN-CTX coherence) across five canonical frequency bands: delta-alpha (2–10 Hz), low-beta (12–20 Hz), high-beta (20–35 Hz), low-gamma (40–70 Hz), and high-gamma (70–100 Hz). This conventional approach computed spectral features by averaging across all neural configurations within each window.

The bradykinesia, dyskinesia, or tremor score at the conclusion of each window served as the dependent variable in each GLM. Hemisphere and participant identity were modelled as categorical covariates to control for inter-hemispheric and inter-individual variability. This modelling framework incorporates patient level heterogeneity through fixed effects, which implicitly adjusts for demographic or clinical variables. p-values for individual predictors were corrected for multiple comparisons using FDR correction, with statistical significance evaluated at an alpha threshold of 0.01. Similar covariates and multiple comparisons correction were applied for all subsequent GLM analyses.

### Modelling symptom severity using HMM derived spatio-spectral features

As per the static spectral modelling, we leveraged a GLM to interrogate the relationship between HMM derived spatio-spectral features and motor symptom severity. For each 2-min-long window, we extracted 15 spatio-spectral features per state, comprising three spatial metrics (STN PSD, CTX PSD, and STN–CTX coherence) across five canonical frequency bands: delta–alpha (2–10 Hz), low-beta (12–20 Hz), high-beta (20–35 Hz), low-gamma (40–70 Hz), and high-gamma (70–100 Hz). Across four states, this yielded 60 predictors.

### Modelling symptom severity using HMM derived temporal features

Finally, GLM analyses were also deployed to probe the relationship between state temporal properties and bradykinesia, dyskinesia, or tremor severity. Given the inherent correlations between temporal metrics across states (e.g., fractional occupancies summing to unity), we fit separate GLMs for each temporal property from each state, to avoid multicollinearity. For each model, the dependent variable was the PKG® symptom score (bradykinesia, dyskinesia or tremor), whilst the independent variable was the temporal feature of interest for each HMM state: fractional occupancy, mean lifetime, visit interval, or switching rate.

### Ethics

Ethical approval was granted by the University of California, San Francisco IRB under a physician sponsored investigational device exemption (IDE), protocol #G180097, and was registered at ClinicalTrials.gov (NCT03582891). Signed informed consent was obtained from all participants.

### Statistics

No a priori sample size estimation was performed due to the restricted availability of investigational RC + S implants and the limited number of patients with simultaneous motor cortex–STN recordings. All available datasets meeting predefined quality criteria (see Methods: Patient Cohort and Electrode Localization; Hidden Markov Model Fit; PKG® Alignment and Preprocessing of Behavioral Data) were included in the analysis. Beyond these criteria (e.g., removal of non-contiguous segments, sleep epochs, and condition-specific PKG® thresholds; see Methods: PKG® Alignment and Preprocessing of Behavioral Data), no additional data exclusion was performed.

### Role of funders

The funders had no role in any aspects of the study design or analysis.

## Results

### Spectral fingerprints of Cortico-Subthalamic states

**Fig. 1:**
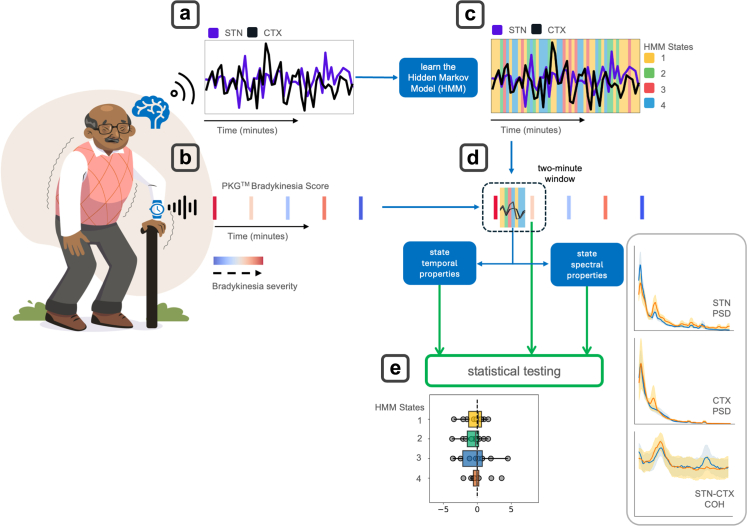
**Overview of the analysis pipeline. (a)** Neural recordings were wirelessly streamed from the subthalamic nucleus (STN) and the ipsilateral motor cortex (CTX) in patients with Parkinson's disease (PD) during naturalistic behavior. **(b)** Concurrent behavioral data were obtained via bradykinesia scores recorded every 2 min using the PKG™ watch contralateral to the hemisphere from which the neural data was streamed. **(c)** A single Hidden Markov Model (HMM) was fitted across the entire dataset, revealing distinct state time courses, representing the probability of occupying specific, statistically defined neural states at each time point. **(d)** For each 2-min window between consecutive bradykinesia scores, spectral and temporal properties were extracted for each HMM state, including power spectral density (PSD) of the STN and CTX, as well as coherence (COH) between STN and CTX. **(e)** The extracted neural features were then regressed onto the subsequent bradykinesia score, enabling an investigation of the mechanistic relationship between dynamic brain state properties and motor impairment with spatial, spectral, and temporal precision.

**Fig. 2:**
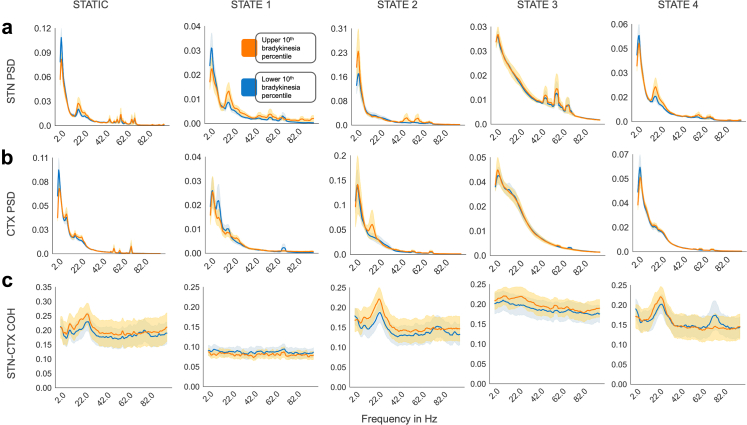
**Distinct spectral fingerprints of HMM states.** To investigate whether each hidden Markov model (HMM) state exhibited unique or redundant spectral responses to bradykinesia, we visualised how spectral properties varied across states at different bradykinesia levels. The power spectral density (PSD) is shown for each state in **(a)** the subthalamic nucleus (STN), **(b)** the motor cortex (CTX), and **(c)** the coherence (COH) between STN and CTX. The first column displays static spectral characteristics computed across the entire dataset without state decomposition, enabling direct comparison between conventional and state-resolved spectral analysis. Spectral properties are compared between the upper 10th percentile and lower 10th percentile of bradykinesia scores. The solid line represents the mean spectra across the 5 participants and 2 hemispheres for each participant, and the shading represents the standard error of the mean across participants and hemispheres. Each state demonstrated a distinct spectral profile in response to bradykinesia. For example, **State 2** exhibited low-gamma power changes in the STN, as well as localized cortical and STN-CTX coherence alterations in the beta range with increasing bradykinesia. In contrast, **State 4** was characterized by local beta power changes in the STN, with only high-gamma coherence scaling inversely with bradykinesia at the circuit level. States 1 and 3 showed relatively flat spectral characteristics in the circuit, with no observable spectral shifts in response to bradykinesia.

We first sought to characterise the spatial and spectral properties of time resolved latent states inferred by the HMM and to compare these with time averaged (static) spectra. Each HMM state captured both local dynamics within individual brain regions (STN and motor cortex) and the coherence patterns between these regions, enabling a comprehensive assessment of both local neural activity and inter-regional connectivity (Fig. 1). Fig. 2 shows the results of spectral analysis for states 1–4, with STN power spectra (Fig. 2a), motor cortical power spectra (Fig. 2b) and STN-motor cortex coherence (Fig. 2c) all displayed. The first column displays the static spectral characteristics of the entire dataset, enabling direct visual comparison between state-resolved and conventional spectral analysis. This comparison demonstrates that the state-resolved approach provides finer resolution about the spatial and temporal properties of cortico-STN network configurations.

To further elucidate the behavioural relevance of these spectral features, we visualized how each state's spectral properties varied with contralateral hemibody bradykinesia severity, which is recognised to be a reliable proxy for medication state^45^ (high bradykinesia scores indicating an off-medication state and vice versa). Given that state-wise spectra were computed over 2-min epochs, each aligned to a bradykinesia score (Methods: PKG® Alignment and Preprocessing of Behavioural Data and Spectral Characterization of HMM States), we stratified the data by bradykinesia severity. Specifically, we averaged spectral features across participants and hemispheres within the top and bottom deciles of the bradykinesia score distribution and visualized both the mean (solid line) and standard error (shaded area) for each condition. The goal of this analysis was to provide a visual intuition demonstrating that spectral characteristics differ between HMM states and systematically vary with bradykinesia severity and medication status.

The spectral architecture of each HMM state revealed distinct local and circuit-level characteristics. State 2 stood out with the highest overall power in both STN and cortical regions, yet this elevated local activity did not translate to enhanced coherence. Instead, states 2–4 all showed comparably strong STN-motor cortex coherence, while state 1 displayed markedly weaker inter-regional coupling.

Beta-band dynamics within the STN showed state-specific patterns. States 1 and 4 both exhibited clear beta peaks that grew more prominent with increasing bradykinesia severity. However, their cortical counterparts behaved differently: state 1 showed declining low–beta activity in motor cortex as bradykinesia worsened, while beta activity in state 4 remained unchanged. State 2 presented a different picture, with a cortical beta peak emerging only during high-bradykinesia periods. This divergence suggests that STN and cortical beta rhythms operate somewhat independently, with symptom sensitivity varying by network state.

Gamma frequency patterns added another layer of complexity. State 3 was distinguished by multiple gamma peaks in the STN that appeared stable regardless of bradykinesia fluctuations. States 1 and 2 also featured high-frequency STN activity, though cortical gamma remained generally subdued across most states. State 1 proved an exception, showing enhanced cortical gamma specifically during periods of reduced bradykinesia.

The coherence spectra unveiled additional state-specific circuit signatures. States 1 and 3 maintained stable broadband coherence that remained consistent across both frequencies and bradykinesia levels. In contrast, states 2 and 4 displayed more dynamic circuit-level behaviour. State 2 featured a prominent high-beta coherence peak that intensified with bradykinesia severity, while state 4 showed a similar beta coherence peak that scaled less with worsening bradykinesia. Interestingly, state 4 exhibited a high-gamma coherence peak which appeared during low-bradykinesia periods, potentially representing a circuit signature of enhanced motor performance.

Taken together, these results delineate four neurophysiological states with distinct spectral and circuit-level fingerprints that scale differentially with bradykinetic severity and medication state. State 1 exhibits local beta power peaks in both STN and motor cortex, with the STN beta peak intensifying during high bradykinesia periods while cortical beta shows the opposite pattern. At the circuit level, STN-cortex coherence remains relatively flat across all frequencies, indicating that beta oscillations in the two regions occur independently rather than synchronously. This configuration suggests functionally decoupled local beta activity without circuit-level coordination.

State 2 displays elevated local power across multiple frequency bands in both regions. STN local power shows increases in low–gamma activity, while cortical local power exhibits elevated beta activity, both scaling with bradykinesia severity. At the circuit level, STN-cortex coherence shows prominent beta-band peaks that intensify during high bradykinesia periods, alongside high-gamma coherence that emerges during low bradykinesia periods. This state thus combines elevated local power with strong circuit-level beta synchronization.

State 3 is characterized by prominent local gamma peaks in the STN that remain stable across bradykinesia levels, while cortical local power shows relatively flat spectral profiles. At the circuit level, STN-cortex coherence remains modest across all frequency bands, with no clear peaks that scale with symptoms. This state appears to reflect STN-specific gamma activity that operates largely independently of both cortical dynamics and symptom fluctuations.

State 4 exhibits local beta peaks in the STN that intensify during high bradykinesia periods, alongside complex cortical patterns where local beta activity emerges primarily during high bradykinesia periods. At the circuit level, STN-cortex coherence shows a beta-band peak that modestly tracks bradykinesia severity, and a distinct high-gamma coherence peak that emerges specifically during low bradykinesia periods. This state thus combines local beta activity with dynamic circuit-level features that include both pathological beta coherence and potentially compensatory high-gamma coherence. These insights provide the foundation for formal statistical testing of the relationship between symptom severity and state-resolved spectral fingerprints in the following section.

### HMM states reveal the dynamics of spectral contributions to bradykinesia

Building upon the characterization of state-specific spectral profiles, we implemented a GLM analysis to separately compare whether spectral features derived from static and HMM based analysis related to bradykinesia severity. For the static spectral analysis, contralateral bradykinesia severity served as the predictor, while independent variables comprised spectral power across five canonical frequency bands (delta-alpha (2–10 Hz), low-beta (12–20 Hz), high-beta (20–35 Hz), low-gamma (40–70 Hz), and high-gamma (70–100 Hz)) in three spatial domains: subthalamic nucleus (STN), motor cortex (CTX), and STN-CTX coherence. This analysis included 523 h (15,692 2-min windows) of data across all studied hemispheres. In our GLM framework, positive regression coefficients indicate that increased neural activity in a given feature predicts worsening bradykinesia, while negative coefficients indicate that increased activity predicts bradykinesia improvement (protective effect).

**Fig. 3:**
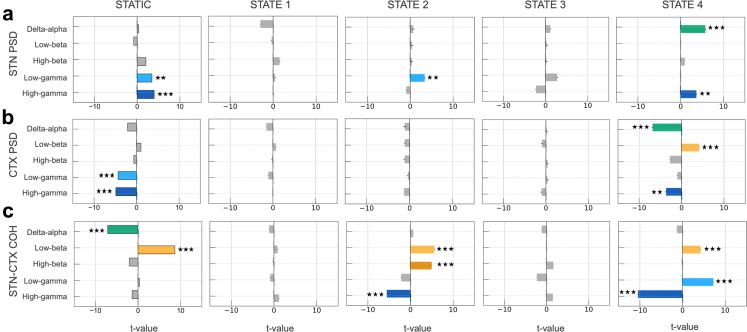
**Mechanistic inference of HMM states in relation to bradykinesia severity.** We applied a generalized linear model (GLM) to test how the spectral properties of each hidden Markov model (HMM) -derived state predicted bradykinesia, as measured by PKG® scores. Bradykinesia severity served as the dependent variable, while predictor variables included power and coherence features from each state, computed over five frequency bands—delta–alpha (2–10 Hz), low-beta (12–20 Hz), high-beta (20–35 Hz), low-gamma (40–70 Hz), and high-gamma (70–100 Hz) at the **(a)** subthalamic nucleus (STN) (power), **(b)** cortex (CTX) (power), and **(c)** STN–cortex (coherence (COH)) levels. Hemisphere and participant identity were included as covariates. There were two hemispheres for each participant. A parallel GLM analysis using conventional static spectral features served as a baseline comparison (displayed in the first column), demonstrating that HMM decomposition revealed mechanistic insights obscured by temporal averaging. Only States 2 and 4 exhibited predictive spectral features. In **state 2**, STN low-gamma power and beta-band coherence predicted worsening bradykinesia severity. In **state 4**, STN delta–alpha and high-gamma power tracked increasing bradykinesia. In motor cortical regions for **state 4**, elevated low-beta power corresponded to worsening bradykinesia, whilst delta–alpha and high-gamma power aligned with bradykinesia ameliorations. At the circuit level, beta coherence again associated with greater impairment, while high-gamma coherence reflected reduced bradykinesia for both **state 2** and **state 4**. The horizontal box plots depict the magnitude of the t-values associated with the predictors. Each colour corresponds to a different frequency band. The black error bars indicate the standard error of mean (SEM) of the t-values. Statistical significance is denoted by (∗∗∗) p < 0.001 and (∗∗) p < 0.01 (adjusted for multiple comparisons using Benjamini Hochberg FDR).

The static spectra model explained 17% of the variance in bradykinesia scores (see left column of Fig. 3). STN gamma activity emerged as a consistent predictor of motor impairment, with both low-gamma (GLM; t-tests on regression coefficients, t (15,478) = 3.46, p < 0.01) and high-gamma (t (15,478) = 3.98, p < 0.001) band activity significantly predicting increased bradykinesia. Motor cortex demonstrated an opposite pattern, with both low-gamma (t (15,478) = −4.37, p < 0.001) and high-gamma (t (15,478) = −4.89, p < 0.001) activity significantly associated with bradykinesia improvement. At the circuit level, delta-alpha coherence showed a significant relationship with bradykinesia improvement (t (15,478) = −7.15, p < 0.001), while low-beta coherence was a significant predictor of symptom worsening (t (15,478) = 8.64, p < 0.001). Neither low nor high beta power at the cortical and STN sites emerged as a significant predictor of bradykinesia severity (STN low beta, t (15,478) = −0.82, p = 0.52; STN high beta, t (15,478) = 2.04, p = 0.07; cortical low beta, t (15,478) = 1.02, p = 0.42; cortical high beta, t (15,478) = −0.66, p = 0.59).

To establish whether HMM states reveal more nuanced spectral contributions, we implemented an analogous GLM using the same frequency bands and spatial domains, decomposed for each of the four HMM states (results presented in Fig. 3). This state-resolved model explained 19% of the variance in bradykinesia scores. More importantly however, only two of the four states–states 2 and 4–exhibited statistically significant associations between their spatio-spectral features and bradykinesia severity.

In state 2, STN low–gamma activity was associated with worsening bradykinesia (GLM; t-tests on regression coefficients, t (15,433) = 3.42, p < 0.01). At the circuit level, beta-band coherence (low-beta: t (15,433) = 5.63, p < 0.001; high-beta: t (15,433) = 5.01, p < 0.001) predicted increased bradykinesia severity, while high-gamma coherence (t (15,433) = −5.60, p < 0.001) was associated with reduced bradykinesia.

In state 4, delta-alpha (t (15,433) = 5.81, p < 0.001) and high–gamma activity (t (15,433) = 3.71, p < 0.01) in the STN predicted increased bradykinesia severity. At the cortical level, elevated low-beta power correlated with worsening bradykinesia (t (15,433) = 4.20, p < 0.001), while delta-alpha (t (15,433) = −6.89, p < 0.001) and high-gamma power (t (15,433) = −3.68, p < 0.01) correlated with motoric improvement. At the circuit-level in state 4, both beta coherence (low-beta: t (15,433) = 4.26, p < 0.001) and low-gamma coherence (t (15,433) = 7.25, p < 0.001) predicted worsening symptoms, whereas high-gamma coherence predicted bradykinesia improvements (t (15,433) = −10.48, p < 0.001). All significant results for the statistical analyses of bradykinesia are presented in Supplementary Table S1.

These results demonstrate that spectral features associated with bradykinesia severity are state-dependent and differ from those identified through conventional static analysis. States 2 and 4 exhibited distinct spectral profiles, with state 2 characterized primarily by circuit-level beta coherence and STN low–gamma associations, while state 4 showed more complex patterns involving both local power features (STN and cortical delta-alpha, cortical beta, and high-gamma) and circuit-level coherence across multiple frequency bands.

### Spectral contributions of HMM states to dyskinesia and tremor

**Fig. 4:**
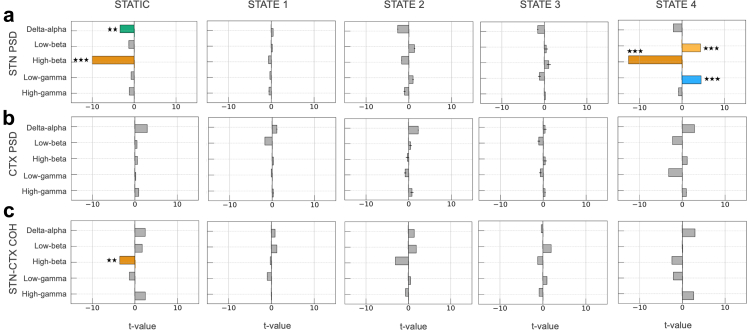
**Mechanistic inference of HMM states in relation to dyskinesia severity.** We used a generalized linear model (GLM) to assess how the spectral properties of each hidden Markov model (HMM)-derived state predicted dyskinesia severity, measured using PKG® scores. Dyskinesia severity served as the dependent variable. Predictor variables included state-specific power and coherence features computed within five frequency bands: delta/alpha (2–10 Hz), low beta (12–20 Hz), high beta (20–35 Hz), low gamma (40–70 Hz), and high gamma (70–100 Hz). These features were evaluated at three levels: **(a)** subthalamic nucleus (STN) power, **(b)** cortical power, and **(c)** STN to cortex coherence. Hemisphere and participant identity were included as covariates, with two hemispheres available for each participant. A parallel GLM analysis based on conventional static spectral features served as a baseline comparison and is shown in the first column. For static spectral analysis, increases in STN delta/alpha and STN high beta power associated with improvements in dyskinesia. At the circuit level, only high beta coherence related to improvements in dyskinesia. Although high gamma coherence had a positive coefficient, neither high nor low gamma power or coherence yielded significant associations with dyskinesia. For the state specific analysis, only STN power in **state 4** showed significant associations with dyskinesia severity. High beta power in the STN was linked to improvements in dyskinesia, whilst both low beta and low gamma power predicted worsening dyskinesia. The horizontal box plots depict the magnitude of the t-values associated with the predictors. Each colour corresponds to a different frequency band. The black error bars indicate the standard error of mean (SEM) of the t-values. Statistical significance is denoted by (∗∗∗) p < 0.001 and (∗∗) p < 0.01 (adjusted for multiple comparisons using Benjamini Hochberg FDR).

We performed parallel GLM analyses to assess how static and HMM state–resolved spectral features relate to dyskinesia and tremor scores. For dyskinesia, a total of 200 (5985 2-min windows) hours of data were analysed (Fig. 4). The static spectral model explained 28.6% of the variance in dyskinesia scores, with delta/alpha (GLM; t-tests on regression coefficients, t (5572) = −3.36, p < 0.01) and high-beta (t (5572) = −10.00, p < 0.001) power within the STN associating with improvements in dyskinesia severity. In addition, high-beta coherence within the motor cortical-STN circuit also predicted dyskinesia improvement (t (5572) = −3.55, p < 0.01).

HMM state resolved GLM analysis explained 30% of the variance in dyskinesia scores. Interestingly, only STN spectral activity within state 4 was predictive of dyskinesia severity. Consistent with the static analysis, STN high–beta activity within this state predicted improvements in dyskinesia (t (5527) = −12.58, p < 0.001). In contrast, both STN low-beta and STN low-gamma power were associated with worsening dyskinesia (low-beta: t (5527) = 4.37, p < 0.001; low-gamma: t (5527) = 4.50, p < 0.001).

**Fig. 5:**
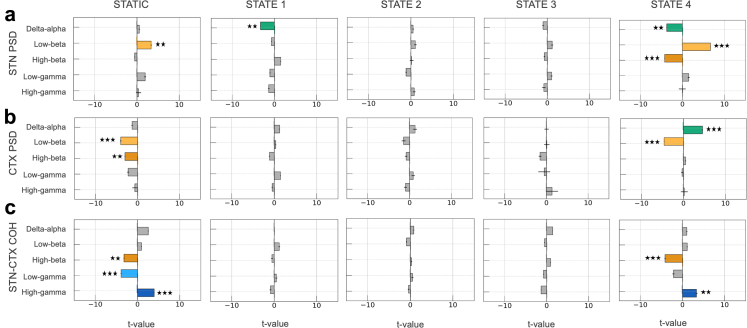
**Mechanistic inference of HMM states in relation to tremor severity.** We used a generalized linear model (GLM) to assess how the spectral properties of each hidden Markov model (HMM)-derived state predicted tremor severity, measured using PKG® scores. Tremor severity served as the dependent variable. Predictor variables included state-specific power and coherence features computed within five frequency bands: delta/alpha (2–10 Hz), low beta (12–20 Hz), high beta (20–35 Hz), low gamma (40–70 Hz), and high gamma (70–100 Hz). These features were evaluated at three levels: **(a)** subthalamic nucleus (STN) power, **(b)** cortical power, and **(c)** STN to cortex coherence. Hemisphere and participant identity were included as covariates, with two hemispheres available for each participant. A parallel GLM analysis based on conventional static spectral features served as a baseline comparison and is shown in the first column. For static spectral analysis, low beta power in the STN predicted worsening tremor. In contrast, both low and high beta power in the cortex predicted tremor improvement. At the circuit level, low gamma coherence and high beta coherence were associated with improvement, whereas high gamma coherence predicted worsening tremor. For the HMM state-based analysis, significant spectral associations were noted for **state 1** and **state 4**. For **state 1**, low delta power in the STN, which did not emerge in the static analysis, associated with improvement in tremor severity. In **state 4**, STN delta/alpha and STN high beta power predicted improvements in tremor, whereas STN low beta power predicted worsening tremor. In the motor cortex, the pattern was reversed: delta/alpha power predicted worsening tremor, and low beta power predicted improvement. At the circuit level, high beta coherence predicted improvement, consistent with the static results, while high gamma coherence predicted worsening tremor, also matching the static analysis. The horizontal box plots depict the magnitude of the t-values associated with the predictors. Each colour corresponds to a different frequency band. The black error bars indicate the standard error of mean (SEM) of the t-values. Statistical significance is denoted by (∗∗∗) p < 0.001 and (∗∗) p < 0.01 (adjusted for multiple comparisons using Benjamini Hochberg FDR).

For the tremor analysis (Fig. 5), we included 84 h of data (2515 2-min windows). The static spectral model explained 35% of the variance in tremor scores and identified STN low–beta activity as a significant tremor predictor (GLM; t-tests on regression coefficients, t (2454) = 3.36, p < 0.01). In contrast, cortical low and high–beta activity was associated with tremor improvement (low-beta: t (2454) = −4.01, p < 0.001; high-beta: t (2454) = −3.01, p < 0.01). Similarly, coherence in the high-beta and low-gamma frequency ranges predicted tremor improvement (high-beta: t (2454) = −3.20, p < 0.01; low-gamma: t (2454) = −3.91, p < 0.001), whereas high-gamma coherence was associated with tremor exacerbation (t (2454) = 3.96, p < 0.001).

The state resolved GLM analysis for tremor, which explained 37% of the variance in tremor scores, again underscored the importance of spectral features within state 4. Within this state STN low-beta power predicted tremor worsening (GLM; t-tests on regression coefficients, t (2409) = 6.67, p < 0.001), whilst STN delta/alpha power and STN high-beta power predicted improvements (delta/alpha: t (2409) = −3.72, p < 0.01; high-beta: t (2409) = −4.23, p < 0.001). At the level of the cortex, these effects were reversed, with delta/alpha power predicting worsening (t (2409) = 4.54, p < 0.001) and low-beta power predicting improvement (t (2409) = −4.60, p < 0.001). Associations between coherence and tremor in state 4 paralleled the findings of the static spectral analysis, with high-gamma coherence predicting worsening (t (2409) = 3.34, p < 0.01) and high-beta coherence predicting improvement (t (2409) = −4.13, p < 0.001). Finally, we observed that increases in STN delta/alpha power in state 1 associated with improvements in tremor severity (t (2409) = −3.29, p < 0.01). All significant results for the statistical analyses of tremor and dyskinesia are presented in Supplementary Tables S2 and S3.

These findings further highlight the utility of state-resolved analysis, with spectral predictors of both dyskinesia and tremor confined to a single state (state 4) characterised by stereotyped patterns of local power and interregional coherence.

### Temporal dynamics reveal a compensatory beta state

A key advantage of HMMs is their capacity to extract both temporal and spatio-spectral properties of latent neural states. We computed temporal metrics for each state (fractional occupancy, mean state lifetime and inter-visit interval) in order to establish their relationship to symptom severity (see Methods: Temporal Metrics of HMM States).

**Fig. 6:**
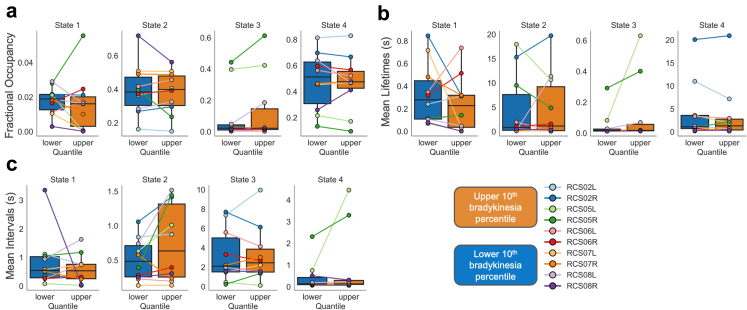
**Temporal dynamics of HMM states in relation to bradykinesia.** In addition to their spectral properties hidden Markov model (HMM) states exhibit distinct temporal characteristics that vary with bradykinesia severity. We quantified three key temporal metrics: (a) **Fractional Occupancy (FO):** The proportion of time each state is active within the dataset. (b) **Mean Lifetime:** The average duration a state remains continuously active before transitioning. (c) **Mean Interval Visit:** The time interval between consecutive activations of the same state. Each metric was computed for all four HMM states, comparing data from the upper and lower 10th percentiles of the bradykinesia score. In the box plots, the line represents the mean and error bars indicate the standard error of the mean (SEM). Individual participant–hemisphere combinations are also shown as connected line plots across the two bradykinesia levels, with colours corresponding to individual participants as detailed in the figure legend. Our findings reveal that the two states exhibiting spectrally distinct circuit-level responses to bradykinesia (**States 2** and **4**) were the most dominant across participants. These states were active for longer durations, persisting from several seconds to tens of seconds. In contrast, the two states that lacked circuit-level spectral responses to bradykinesia (**States 1** and **3**) were short-lived (hundreds of milliseconds) and less prevalent across participants.

Interestingly, states 2 and 4, which previously exhibited distinct coherence signatures in the spectral domain, demonstrated the highest fractional occupancies, indicating that these two network configurations dominated the neural time series relative to states 1 and 3 (Fig. 6a). In parallel, these same states (2 and 4) also displayed prolonged average lifetimes (Fig. 6b). Notably, only state 3 exhibited extended inter-visit intervals, implying more sporadic activation (Fig. 6c). Individual participants displayed idiosyncratic trends in temporal features ordered by the upper and lower bradykinesia deciles, which serve as a proxy for medication state.

**Fig. 7:**
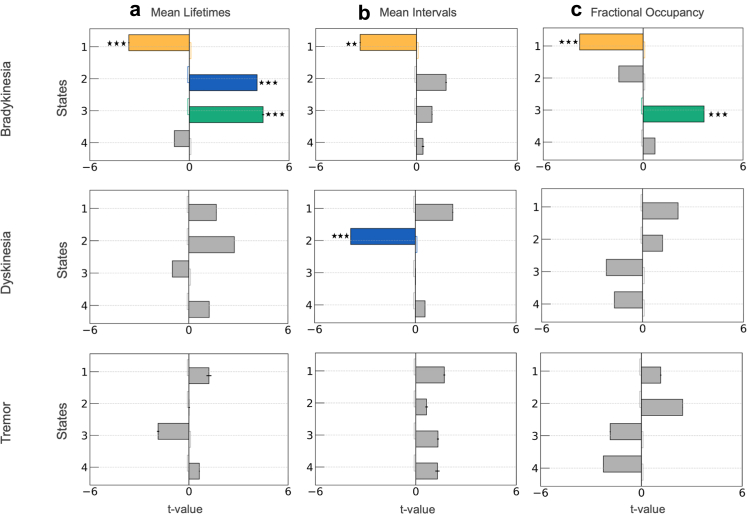
**Temporal metrics of HMM-derived neural states as predictors of bradykinesia, dyskinesia and tremor severity.** A generalized linear model (GLM) framework was used to evaluate associations between temporal characteristics of latent neural states and symptom severity, as indexed by PKG® scores. Bradykinesia, dyskinesia, and tremor were modelled separately as dependent variables. Independent variables comprised state-specific temporal metrics: fractional occupancy, mean lifetime, and mean inter-visit interval. Each temporal metric was assessed in a separate GLM. For bradykinesia, fractional occupancy, mean lifetime, and mean inter-visit interval of state 1 showed significant associations with symptom scores, while temporal metrics of states 2 and 3 were also significantly associated with bradykinesia. For dyskinesia, mean inter-visit interval of state 2 was significantly associated with symptom severity. For tremor, no temporal metric showed a significant association with symptom severity. The horizontal box plots depict the magnitude and signage of the t-values associated with the predictors. Each colour corresponds to a different state. The black error bars at the end of the bar plots depict the standard error of mean (SEM) of the t-values. Statistical significance is denoted by (∗∗∗) p < 0.001 and (∗∗) p < 0.01 (adjusted for multiple comparisons using Benjamini Hochberg FDR).

GLM analysis (see Methods: Modelling Bradykinesia via Temporal Features) revealed that state 1 exhibited temporal properties consistently associated with improved motor function. Specifically, increased fractional occupancy (GLM; t-tests on regression coefficients, t (15,492) = −3.81; p-value < 0.001) and prolonged lifetimes of state 1 (GLM; t-tests on regression coefficients, t (15,489) = −3.65; p-value < 0.001) were significantly associated with reduced bradykinesia severity, suggesting that greater temporal persistence of this state predicts motor improvement (Fig. 7a, c). Longer inter-visit intervals for state 1 were also linked to bradykinesia relief (t (15,489) = −3.36; p-value < 0.01), indicating that temporally dispersed, but sustained re-engagements with this state may be beneficial (Fig. 7b). Importantly, although state 1's spectral features were not directly predictive of bradykinesia, it did exhibit peaks of beta activity both within the cortex and within the STN (Fig. 2a, b; state 1) that were modulated by symptom severity and not associated with cortico-STN beta coherence peaks (Fig. 2c).

Our analysis also revealed that increased fractional occupancy (t (15,492) = 3.66; p-value < 0.001) and prolonged lifetimes (t (15,489) = 4.44; p-value < 0.001) of state 3 were both associated with greater bradykinesia severity (Fig. 7a, c). Interestingly, this effect occurred in the absence of a correlation between state 3's spectral features and bradykinesia, and despite there being no observable scaling of this state's spectral profile with bradykinesia severity across deciles. Additionally, we also observed that increased lifetimes of state 2—a state that predominantly exhibited spectral features correlating with worsening bradykinesia—were associated with increased bradykinesia severity (t (15,489) = 4.07; p-value < 0.001).

No state exhibited temporal properties that were significantly related to tremor severity. For dyskinesia however, longer inter-visit intervals of state 2 associated with lower dyskinesia severity (GLM; t-tests on regression coefficients, t (2465) = −3.85; p-value < 0.001). This result indicates that more frequent recurrence of state 2 was associated with higher dyskinesia scores.

Collectively we conclude that temporal dynamics of latent brain states are important predictors of motor impairment, particularly bradykinesia. Although states 2 and 4 are temporally dominant, it is predominantly their internal spectral features that associate with symptom severity. State 1, which is characterised by short-lived STN and motor cortical beta activity, may represent a compensatory motoric configuration that ameliorates bradykinesia. This suggests that relief from bradykinesia may depend not only on suppressing pathological states, but also on increasing the occurrence of compensatory configurations.

## Discussion

We leveraged Hidden Markov Models (HMMs) to discover latent neural states of cortical and subthalamic nucleus (STN) activity in patients with Parkinson's disease (PD). Our dataset of continuous recordings, acquired while patients took their regular medication during daily activities, allowed us to capture naturalistic fluctuations in medication state—including both on and off medication periods, as well as the substantial variability that exists between these extremes. By relating the identified neural states to dynamic measurements of bradykinesia, dyskinesia and tremor, we highlight that motor symptoms are encoded through both the spectral and temporal properties of states of cortico-STN network activity. Spectral associations with symptom severity were found to be state specific, speaking to the utility of moving beyond single site and single frequency band biomarkers (e.g., STN beta activity) towards a more nuanced understanding of how moment-to-moment fluctuations in brain network dynamics underpin PD pathophysiology.

A key finding of this work is the existence of a compensatory state characterised by short-lived, but uncoupled STN and cortical beta activity that associates with ameliorations of bradykinesia. This compensatory state could represent a potential target state for neuromodulation therapies, and its presence is supported by previous studies demonstrating that the occurrence of short-lived bursts of beta activity within the STN can be associated with improved motor performance.^50^ Moreover, adaptive DBS approaches which act over slower timescales to potentially preserve physiological levels of beta within motor circuits could be more effective than approaches which seek to rapidly terminate beta activity.^51^

Beta oscillations (13–30 Hz) have been a central focus in PD research, due in large part to their consistent elevation in the STN and cortex under dopamine-depleted conditions.^7^, ^8^, ^9^ Increased STN beta power has been linked to bradykinesia and rigidity, prompting clinical strategies such as beta-guided adaptive DBS, that aim to reduce beta amplitude.^52^ Our findings nuance this established paradigm by demonstrating that STN beta power alone was not an independent predictor of bradykinesia severity. Instead, beta-related associations with bradykinesia emerged most strongly through circuit-level features, especially beta-band coherence between the STN and motor cortex. This distinction underscores the importance of network synchrony over local oscillatory magnitude in driving bradykinesia. For tremor, distinct effects were observed such that STN beta activity predicting tremor worsening, whereas increases in beta coherence and cortical beta activity predicted tremor improvement. Finally, in keeping with previous reports, we observed that STN high beta power and coherence predicted dyskinesia improvement.^36^

A more granular analysis of the state-specific data revealed that cortical beta activity had a greater predictive influence on bradykinesia than STN beta power in certain HMM states. For example, in state 4, low–beta activity in the motor cortex emerged as a robust predictor of bradykinesia severity, whereas in other states (e.g., state 2), neither STN nor cortical beta power significantly contributed to bradykinesia. Notably, the associations between beta activity and both tremor and dyskinesia identified in the static analysis were likewise restricted to state 4, further underscoring the importance of this network configuration for motor function.

State 1 offered additional insights, particularly with respect to temporal dynamics. State 1 exhibited a distinct beta peak within the STN across both low and high bradykinesia deciles, with a slightly lower frequency beta peak localized to the motor cortex only in the low bradykinesia decile. Spectrally, state 1 exhibited a relatively flat profile of circuit level coherence. Although the spectral features of state 1 were not associated with bradykinesia, increased temporal occurrence of state 1 was predictive of bradykinesia ameliorations.

Taken together, these findings underscore the state dependent role of beta oscillations, wherein they are not inherently detrimental, but become pathologically relevant when occurring within certain temporally segregated network configurations.

A growing literature frames gamma–band activity (50–100 Hz) as broadly prokinetic in PD, particularly because it is enhanced by dopaminergic medications and correlates with improved movement speed.^39^, ^40^, ^41^, ^42^ Our results generally support this prokinetic narrative for STN–cortical high gamma coherence (70–100 Hz), yet they also highlight a crucial caveat: local gamma oscillations within the STN can, in certain states, be associated with worsening bradykinesia as well as with the hyperkinetic state of dyskinesia (Fig. 5a; state 4). Notably, the association between STN gamma and dyskinesia emerged only in the state-based analysis and was not evident in the static spectral analysis. This contrast underscores the multifaceted role of gamma band oscillations, whereby their functional impact depends on: (1) the site where gamma emerges (cortex versus STN), (2) whether it is expressed coherently at the circuit level or remains an isolated local burst, and (3) the nature of lower frequency oscillations that emerge as significant predictors in the same spatial location.

In state 4, local STN high-gamma power scaled positively with bradykinesia severity. One consistent pattern was the shared signage of high-gamma and low-frequency (delta–alpha, ∼2–10 Hz) features in our regression models. That is, when STN high-gamma increased in these states, STN delta-alpha power rose in parallel, and both predicted greater motoric impairment (Fig. 3a; state 4). Our data revealed contrasting results for the motor cortex in state 4, where increased local cortical high-gamma and delta-alpha activity were associated with reduced bradykinesia scores (Fig. 3b; state 4). These opposing directionalities of delta–alpha effects in STN versus cortex argue against a common far-field source and support the interpretation that these signals reflect regionally distinct, locally generated activity.

Additionally, the relationship between low frequency delta/alpha oscillations and gamma, and their shared differential impact on bradykinesia at the cortical and STN sites, raises the possibility of local non-linear mechanisms for propagating gamma oscillations.^28^^,^^53^, ^54^, ^55^, ^56^, ^57^ For example, high gamma in the STN could be phase locked to or entrained by slower local STN rhythms often associated with akinetic or low dopaminergic states.^33^ Such coupling could neutralize or reverse gamma's usual prokinetic advantage by aligning it with activity that can perpetuate bradykinesia.

STN–cortical high gamma coherence exerted prokinetic influences across states in our data. This distinction highlights that gamma coherence, rather than local gamma amplitude alone may be the key signal for binding cortex and STN into a functional network that can initiate movements efficiently. Prior work^40^^,^^41^ has shown that dopaminergic medication and deep brain stimulation enhance cortico-subthalamic gamma coupling at movement onset, particularly during faster or larger-amplitude movements. Our findings bolster that narrative but add nuance by showing how coherence can remain prokinetic (Fig. 3b; state 2) even when local STN gamma amplitude might be anti-kinetic (Fig. 3a; state 2). Functionally, this suggests that the network requires high-frequency communication channels to bypass or counteract pathological beta synchrony; in the absence of that coherent linkage, local STN gamma by itself does not guarantee movement facilitation. Notably, gamma coherence also increased with tremor severity in state 4, suggesting that gamma coherence may need to be maintained within a narrow dynamic range to ameliorate bradykinesia without inadvertently promoting tremor.

Taken together, our observations underscore that STN beta amplitude alone is an insufficient biomarker of bradykinesia, although it reliably tracks changes in the severity of tremor and dyskinesia in a state-specific manner. By contrast, beta-band coherence appears to provide a more reliable index of bradykinesia severity. Consequently, therapies that aim solely to suppress beta power may yield inconsistent outcomes if they ignore underlying circuit configurations and inadvertently suppress compensatory beta states associated with bradykinesia amelioration. State-aware neuromodulation could instead selectively target neural states linked to an individual patient's dominant symptoms at a given time. For example, targeting beta activity in state 4 during periods of exaggerated tremor may be beneficial, whereas shifting stimulation to states in which beta coherence is most detrimental could enable a more precise and adaptive approach to bradykinesia relief.^58^, ^59^, ^60^, ^61^

Similarly, for gamma oscillations, interventions that non-specifically enhance gamma (e.g., via transcranial stimulation^39^^,^^62^^,^^63^) could yield inconsistent results if they do not target coherent gamma bursts or inadvertently exacerbate local STN gamma tied to both bradykinesia and dyskinesia. Looking ahead, adaptive DBS systems might deliver stimulation only when gamma coherence dips or when local STN gamma uncouples from cortical input. Such closed-loop methods could selectively reinforce the circuit-level gamma that has prokinetic effects. Future neuromodulation strategies might dynamically track multiple spectral and temporal features (rather than a single beta amplitude threshold) to disrupt harmful synchrony while sparing or fostering beneficial oscillatory states.

Several study limitations merit consideration. Firstly, we utilized a four-state HMM, selected for its balance of interpretability and explanatory power. However, this may underrepresent the full spectrum of dynamic states present in the data. Future models could explore larger or hierarchical state architectures or incorporate explicit duration modelling to capture the persistence of pathological versus compensatory states. Secondly, while PKG® sensors provided continuous, ecologically valid measures of motor function, their resolution is limited compared to high-density motion capture or wearable kinematic systems. This constraint may obscure finer resolution motor fluctuations and their alignment with neural states. Thirdly, our analysis focused exclusively on three key motor symptoms and did not include non-motor symptoms, which may also influence cortico-STN oscillatory states. However, we note that HMMs are well suited to identifying both motor and non-motor states,^33^^,^^64^ suggesting that future studies could leverage these methods to investigate non-motor symptoms in PD. Fourthly, the correlational nature of our analysis limits causal interpretation. Real-time, state-triggered neuromodulation experiments will be required to determine how stimulation alters neural states over time and across disease progression, and to establish their causal role in specific PD symptoms.

Our study includes a small cohort of five patients (10 hemispheres), which reflects the restricted availability of investigational RC + S implants and the relative scarcity of simultaneous motor cortex–STN recordings in humans.^45^ This sample size warrants careful consideration of sample representativeness and statistical power. Several factors support the robustness of our findings. First, intensive within-subject sampling provided substantial statistical power, with approximately 31,380 observations derived from 1046 h of recordings constituting the unit of analysis rather than the number of patients. Second, the within-subject design—where each patient serves as their own control across brain states—mitigates multiple sources of between-subject variability that would require much larger samples in between-subject designs. Third, training a single group-level HMM across all patients ensured that identified states represent recurring network configurations shared across individuals rather than patient-specific patterns. While future studies with larger cohorts will be important for characterising inter-individual variability and refining population–level parameters, our results provide a foundation for understanding the multidimensional neural architecture underlying motor symptoms in Parkinson's disease.

Our study provides high spatial and temporal resolution insights into the link between cortico-STN network activity and motor symptoms in PD. By modelling brain activity as transitions between statistically defined states with distinct spectral and coherence profiles, we capture a dynamic view of symptom expression that enables us to move beyond time-averaged biomarkers. This state-based approach could offer practical pathways toward adaptive, precision-guided interventions that respond to how the brain organizes its own activity in real time.

## Contributors

Conceptualization, A.S., W.N., S.L., P.S., A.O.; Methodology, A.S., A.H., M.S., S.L., P.S., A.O.; Software & Formal Analysis, A.S., T.L., B.A.; Data Curation, A.S., A.H., M.S., W.N., S.L., P.S., A.O.; Supervision, S.L., P.S., A.O.; Funding Acquisition, S.L., P.S., A.O.

A.S., T.L., B.A., and A.O., accessed and verified the underlying data.

All authors read and approved the final version of the manuscript.

## Data sharing statement

Due to the sensitive nature of the data, patient-level datasets cannot be made publicly available. Fully anonymised data generated in this study will be made available to qualified researchers upon reasonable request, subject to a data sharing agreement, via the MRC CoRE in Restorative Neural Dynamics data platform (https://data.mrc.ox.ac.uk/).

Code for implementation of this manuscript is available at: https://github.com/saltwater-tensor/PD-neural-dynamics-motor-states.

## Declaration of interests

P.S. is a consultant for Echo Neurotechnologies and InBrain Neuroelectronics. S.L. is a consultant for Iota Biosciences and has received grant funding from the National Institutes of Health (UG3NS140730 and R01NS131405). S.L. is the co-founder and CEO of Ocean Neuro. University of California, San Francisco (UCSF) owns patents that have come from the S.L.’s lab in neural sensing and adaptive DBS. W.N. is a consultant for InBrain Neuroelectronics. The other authors declare no competing interests.
