# Supplementary material for: Dynamic neural states underpin motor symptom severity in Parkinson's disease: a longitudinal analysis of chronic cortico-subthalamic nucleus recordings
Source: eBioMedicine. 2026 May 12;128:106293. doi: 10.1016/j.ebiom.2026.106293 (PMC13191631; doi:10.1016/j.ebiom.2026.106293)
Supplement: Supplementary Materials [file mmc1.pdf]

Dynamic neural states underpin motor symptom severity in Parkinson's disease: a longitudinal analysis of chronic cortico-subthalamic nucleus recordings

Abhinav Sharma, Tao Liu, Bahman Abdi-Sargezeh, Amelia Hahn, Maria Shcherbakova, Wolf-Julian Neumann, Simon Little, Philip Starr, Ashwini Oswal

Contents

Supplementary Methods 2

Streaming of DBS Device Data 2

Supplementary Results 2

Supplementary Figures 3

References 5

Supplementary Tables 6

Supplementary Methods

Streaming of DBS Device Data

The RC+S device employs a User Datagram Protocol (UDP) to transmit data to an external tablet in packets (average duration 50 ms). Occasionally (1-5%), packets are dropped or lost in transmission e.g., when the patient walks out of the 12m range of the recording tablet. As we did not interpolate dropped packets, but removed them, recordings were divided into multiple sessions. Freely available software was used to account for dropped packets (https://github.com/openmind-consortium/Analysis-rcs-data)[1].

Supplementary Results

In a separate analysis, we examined how the spectral properties of HMM states differed between the physiological states of wakefulness and sleep. Periods of sleep were identified using immobility as a surrogate marker, defined as at least 2 minutes of immobility with a bradykinesia score exceeding 80[2,3]. As shown in **Supplementary Figure 2**, state-specific spectra captured meaningful physiological differences between wakefulness and sleep.

The most pronounced modulation was observed at the circuit level, with increased cortico–STN coherence during sleep. In addition, beta peaks that were prominent in States 2 and 4 during wakefulness were attenuated during sleep, while lower-frequency oscillations became more pronounced. Finally, power spectra for States 3 and 4 exhibited increased low-frequency activity in both the STN and motor cortex during sleep. Together, these findings demonstrate that the spectral signatures of HMM states sensitively reflect transitions between distinct physiological states.

Supplementary Figures

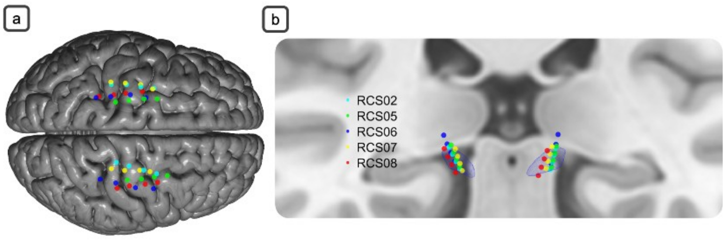

Supplementary Figure 1. Electrode contact locations. (**a**) The left image shows cortical contact locations, superimposed on a cortical mesh derived from a template MRI in Montreal Neurological Institute (MNI) space. (**b**) The right image shows STN electrode contact locations for all 5 patients, superimposed on an STN mesh (blue) and the same template MRI (coronal view).

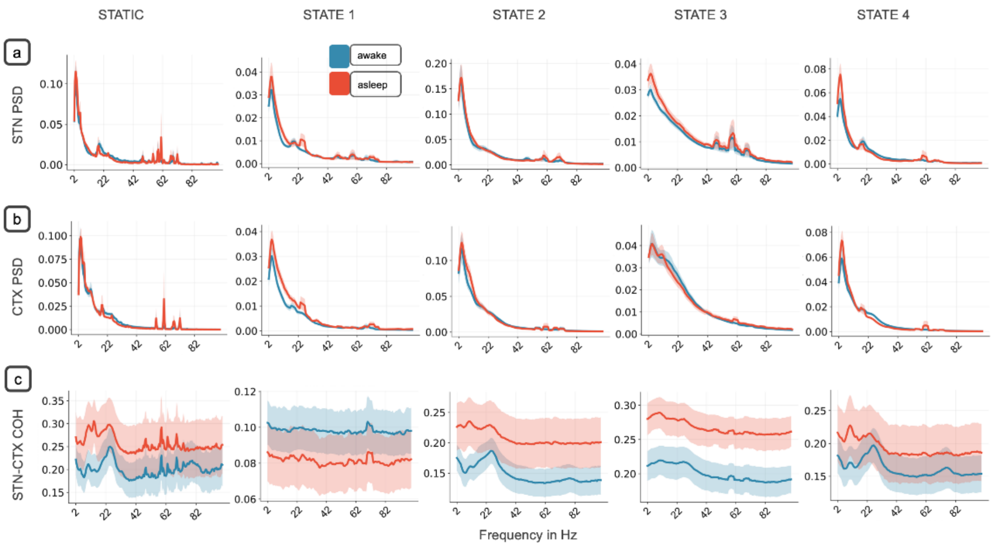

Supplementary Figure 2. Distinct spectral fingerprints of HMM states during wakefulness and sleep. To assess whether the spectral features identified in the HMM states and static analyses reflect meaningful physiological processes, we compared spectral profiles during awake and asleep periods. The awake condition encompasses a range of behavioural states, including epochs of bradykinesia, natural movement, dyskinesia, and tremor. Despite this heterogeneity, clear differences emerged between wakefulness and sleep. The most pronounced modulation was observed at the circuit level, particularly in cortico–STN coherence. During sleep, the beta peaks prominent in States 2 and 4 during wakefulness were attenuated, while lower-frequency oscillations became more prominent. Overall cortico–STN coherence was increased in the asleep condition. In addition, power spectra for States 3 and 4 showed increased low-frequency activity in both the STN and motor cortex during sleep. Together, these observations demonstrate that the spectral signatures of HMM states capture meaningful differences between physiological states.

References

1. Gilron R, Little S, Perrone R, et al. Long-term wireless streaming of neural recordings for circuit discovery and adaptive stimulation in individuals with Parkinson’s disease. *Nat Biotechnol*. 2021;39(9):1078-1085.

2. Olaru M, Cernera S, Hahn A, et al. Motor network gamma oscillations in chronic home recordings predict dyskinesia in Parkinson’s disease. *Brain*. 2024;147(6):2038-2052.

3. Yao P, Sharma A, Abdi-Sargezeh B, et al. Beta Burst Characteristics and Coupling within the Sensorimotor Cortical-Subthalamic Nucleus Circuit Dynamically Relate to Bradykinesia in Parkinson’s Disease. *Movement Disorders*. 2025;40(5):962-968.

Supplementary Tables

Supplementary Table 1. Summary of statistically significant associations between neural features and bradykinesia severity

Analysis Type	Neural Feature	Spatial Location	Direction of Effect	t-statistic	p-value	Figure Reference
**Static Spectral Analysis**						
	Low-gamma power	STN	Worsening	3.46	<0.01	Fig 3a, Static
	High-gamma power	STN	Worsening	3.98	<0.001	Fig 3a, Static
	Low-gamma power	Cortex	Improvement	-4.37	<0.001	Fig 3b, Static
	High-gamma power	Cortex	Improvement	-4.89	<0.001	Fig 3b, Static
	Delta-alpha coherence	STN-Cortex	Improvement	-7.15	<0.001	Fig 3c, Static
	Low-beta coherence	STN-Cortex	Worsening	8.64	<0.001	Fig 3c, Static
**State-Specific Spectral Analysis**						
State 2	Low-gamma power	STN	Worsening	3.42	<0.01	Fig 3a, State 2
State 2	Low-beta coherence	STN-Cortex	Worsening	5.63	<0.001	Fig 3c, State 2
State 2	High-beta coherence	STN-Cortex	Worsening	5.01	<0.001	Fig 3c, State 2
State 2	High-gamma coherence	STN-Cortex	Improvement	-5.60	<0.001	Fig 3c, State 2
State 4	Delta-alpha power	STN	Worsening	5.81	<0.001	Fig 3a, State 4
State 4	High-gamma power	STN	Worsening	3.71	<0.01	Fig 3a, State 4
State 4	Delta-alpha power	Cortex	Improvement	-6.89	<0.001	Fig 3b, State 4
State 4	Low-beta power	Cortex	Worsening	4.20	<0.001	Fig 3b, State 4
State 4	High-gamma power	Cortex	Improvement	-3.68	<0.01	Fig 3b, State 4
State 4	Low-beta coherence	STN-Cortex	Worsening	4.26	<0.001	Fig 3c, State 4
State 4	Low-gamma coherence	STN-Cortex	Worsening	7.25	<0.001	Fig 3c, State 4
State 4	High-gamma coherence	STN-Cortex	Improvement	-10.48	<0.001	Fig 3c, State 4
**Temporal Analysis**						
State 1	Fractional occupancy	-	Improvement	-3.81	<0.001	Fig 7c
State 1	Mean lifetime	-	Improvement	-3.65	<0.001	Fig 7a
State 1	Mean interval	-	Improvement	-3.36	<0.01	Fig 7b
State 2	Mean lifetime	-	Worsening	4.07	<0.001	Fig 7a
State 3	Fractional occupancy	-	Worsening	3.66	<0.001	Fig 7c
State 3	Mean lifetime	-	Worsening	4.44	<0.001	Fig 7a

Supplementary Table 2. Summary of statistically significant associations between neural features and dyskinesia severity

Analysis Type	Neural Feature	Spatial Location	Direction of Effect	t-statistic	p-value	Figure Reference
**Static Spectral Analysis**						
	High-beta power	STN	Improvement	-10.00	<0.001	Fig 4a, Static
	Delta-alpha power	STN	Improvement	-3.36	<0.01	Fig 4a, Static
	High-beta coherence	STN-Cortex	Improvement	-3.55	<0.01	Fig 4c, Static
**State-Specific Spectral Analysis**						
State 4	Low-beta power	STN	Worsening	4.37	<0.001	Fig 4a, State 4
State 4	High-beta power	STN	Improvement	-12.58	<0.001	Fig 4a, State 4
State 4	Low-gamma power	STN	Worsening	4.50	<0.001	Fig 4a, State 4
**Temporal Analysis**						
State 2	Mean interval	-	Improvement	-3.85	<0.001	Fig 7b

Supplementary Table 3. Summary of statistically significant associations between neural features and tremor severity

Analysis Type	Neural Feature	Spatial Location	Direction of Effect	t-statistic	p-value	Figure Reference
**Static Spectral Analysis**						
	Low-beta power	STN	Worsening	3.36	<0.01	Fig 5a, Static
	Low-beta power	Cortex	Improvement	-4.01	<0.001	Fig 5b, Static
	High-beta power	Cortex	Improvement	-3.01	<0.01	Fig 5b, Static
	High-beta coherence	STN-Cortex	Improvement	-3.20	<0.01	Fig 5c, Static
	Low-gamma coherence	STN-Cortex	Improvement	-3.91	<0.001	Fig 5c, Static
	High-gamma coherence	STN-Cortex	Worsening	3.96	<0.001	Fig 5c, Static
**State-Specific Spectral Analysis**						
State 1	Delta-alpha power	STN	Improvement	-3.29	<0.01	Fig 5a, State 1
State 4	Delta-alpha power	STN	Improvement	-3.72	<0.01	Fig 5a, State 4
State 4	Low-beta power	STN	Worsening	6.67	<0.001	Fig 5a, State 4
State 4	High-beta power	STN	Improvement	-4.23	<0.001	Fig 5a, State 4
State 4	Delta-alpha power	Cortex	Worsening	4.54	<0.001	Fig 5b, State 4
State 4	Low-beta power	Cortex	Improvement	-4.60	<0.001	Fig 5b, State 4
State 4	Delta-alpha coherence	STN-Cortex	Improvement	-4.13	<0.001	Fig 5c, State 4
State 4	High-gamma coherence	STN-Cortex	Worsening	3.34	<0.01	Fig 5c, State 4
